# Suspension of Dental Journals

**Published:** 1899-01

**Authors:** 


### SUSPENSION OF DENTAL JOURNALS.

It is not a matter of surprise to be obliged to record the discontinuance of a journal, for these periodicals rise and fall with a regularity quite equalling the appearance of new candidates for professional favor; but in the past few weeks dentistry has lost two notable journals, the departure of which from our exchanges is a cause of regret. The two are the *American Dental Weekly*, published at Atlanta, Ga., and the *Dental Practitioner*, of Buffalo, N. Y.

It was a brave effort of Dr. Catching and his co-workers to establish a weekly journal, and it deserved a better fate. While it lasted it was ably edited and did an excellent work. The time has not arrived, however, when a weekly dental journal can be sustained. The amount of labor devoted to a monthly periodical is quite as much as the average professional man can bear, and until the compensation will warrant an entire giving up of other duties such a journal cannot be continued. It is a question whether, even then, weekly journals will be required.

The *Dental Practitioner and Advertiser* was issued but four times a year. This is the other extreme; but, notwithstanding this, the numbers were always eagerly read by a large circle, and its editorials exerted a greater influence than, possibly, any other journal. The editor, Dr. Barrett, wielded a virile pen, not always according to our ideas, but with a force that commanded attention. It is with an expression of more than the usual regret that he has been obliged to leave the editorial chair; but the hope is indulged that in some way his valuable and always energetic thought may find an avenue of expression. His contemporaries will, we are sure, agree with the opinion that this should be in the same line of work; for it is here that he is peculiarly fitted by experience and ability to lead his profession to more correct thinking and practice.

